# Supplementary material for: Aberrant non-canonical NF-κB signalling reprograms the epigenome landscape to drive oncogenic transcriptomes in multiple myeloma
Source: Nat Commun. 2024 Mar 21;15:2513. doi: 10.1038/s41467-024-46728-4 (PMC10957915; doi:10.1038/s41467-024-46728-4)
Supplement: Supplementary file 3 — Reporting Summary [file 41467_2024_46728_MOESM3_ESM.pdf]

nature portfolio

Corresponding author(s): Yinghui Li

Last updated by author(s): Mar 6, 2024

Reporting Summary

Nature Portfolio wishes to improve the reproducibility of the work that we publish. This form provides structure for consistency and transparency in reporting. For further information on Nature Portfolio policies, see our Editorial Policies and the Editorial Policy Checklist.

Statistics

For all statistical analyses, confirm that the following items are present in the figure legend, table legend, main text, or Methods section.

n/a	Confirmed	
☐	☒	The exact sample size (*n*) for each experimental group/condition, given as a discrete number and unit of measurement
☐	☒	A statement on whether measurements were taken from distinct samples or whether the same sample was measured repeatedly
☐	☒	The statistical test(s) used AND whether they are one- or two-sided<br>*Only common tests should be described solely by name; describe more complex techniques in the Methods section.*
☐	☒	A description of all covariates tested
☐	☒	A description of any assumptions or corrections, such as tests of normality and adjustment for multiple comparisons
☐	☒	A full description of the statistical parameters including central tendency (e.g. means) or other basic estimates (e.g. regression coefficient) AND variation (e.g. standard deviation) or associated estimates of uncertainty (e.g. confidence intervals)
☐	☒	For null hypothesis testing, the test statistic (e.g. *F*, *t*, *r*) with confidence intervals, effect sizes, degrees of freedom and *P* value noted<br>*Give P values as exact values whenever suitable.*
☒	☐	For Bayesian analysis, information on the choice of priors and Markov chain Monte Carlo settings
☒	☐	For hierarchical and complex designs, identification of the appropriate level for tests and full reporting of outcomes
☒	☐	Estimates of effect sizes (e.g. Cohen's *d*, Pearson's *r*), indicating how they were calculated

Our web collection on statistics for biologists contains articles on many of the points above.

Software and code

Policy information about availability of computer code

Data collection	qPCR: CFX Maestro Software<br>Western blots/gel images: Image Lab/ImageJ<br>Bioanalyser: Agilent 2100 Bioanalyzer software<br>FACS (apoptosis/proliferation): BD FACSDiva™ / Flowjo<br>Colorimetric analysis (adhesion assay): Tecan icontrol
Data analysis	#bash<br>bedtools 2.30.0<br>bowtie2 2.3.4.3<br>bwa 0.7.17-r1198-dirty<br>cooler 0.8.11<br>cutadapt 2.5<br>deeptools 3.3.1<br>FitHiChIP 9.1<br>gatk 4.3.0.0<br>hicexplorer 3.7.1<br>macs2 2.1.2<br>picard 2.20.7<br>pygenometracks 3.6<br>ROSE 1.3.0<br>samblaster 0.1.24

samtools ≥1.12
tobias 0.13.3
trimmomatic 0.39
#R
biomaRt 2.50.0
circlize 0.4.15
clusterProfiler 4.2.0
ComplexHeatmap 2.10.0
cowplot 1.1.1
DESeq2 1.34.0
DiffBind ≥2.10
diffloop 1.20.0
DOSE 3.20.0
edgeR 3.36.0
eulerr 6.1.1
factoextra 1.0.7
ggalluvial 0.12.3
ggplot2 3.3.5
ggrepel 0.9.1
GO.db 3.14.0
GOSemSim 2.20.0
IHW 1.22.0
limma 3.50.1
rGREAT 1.26.0
tidyverse 1.3.1

For manuscripts utilizing custom algorithms or software that are central to the research but not yet described in published literature, software must be made available to editors and reviewers. We strongly encourage code deposition in a community repository (e.g. GitHub). See the Nature Portfolio guidelines for submitting code & software for further information.

Data

Policy information about availability of data

All manuscripts must include a data availability statement. This statement should provide the following information, where applicable:

- Accession codes, unique identifiers, or web links for publicly available datasets
- A description of any restrictions on data availability
- For clinical datasets or third party data, please ensure that the statement adheres to our policy

Cell line p52 KD RNA-seq (KMS-11 and MM1.144), p52 KD ATAC-seq (KMS-11), p52 KD H3K27ac HiChIP (KMS-11), p52 KD H3K27ac ChIP-seq (KMS-11, MM1.144 and LP1), endogenous H3K27ac and p52 ChIP-seq (KMS-11, JJN3, LP1, MM1.S, U266, MM1.144) raw and processed data generated in this study as well as the RNA-seq derived from NUHS myeloma patients have been deposited in the GEO database under accession code GSE230526.
All MMRF CoMMpass data can be accessed at https://research.themmrf.org/
All public epigenomic data was sourced from the following studies:
- Ordoñez et al., 2020: http://resources.idibaps.org/paper/chromatin-activation-as-a-unifying-principle-underlying-pathogenic-mechanisms-in-multiple-myeloma
- Alvarez-Benayas et al., 2021: ftp://ftp.ebi.ac.uk/pub/databases/blueprint/releases/20160816/homo_sapiens
- Jin et al., 2018: PRJEB25605
- Jia et al., 2021: PRJNA608681

Research involving human participants, their data, or biological material

Policy information about studies with human participants or human data. See also policy information about sex, gender (identity/presentation), and sexual orientation and race, ethnicity and racism.

Reporting on sex and gender	The detailed clinical characteristics including sex were obtained from patients' health records and previously published in supplemental table 1 in (Jia et al., 2021). Sex and gender were not considered in the study design as multiple myeloma progression has not been reported to be influenced by either.
Reporting on race, ethnicity, or other socially relevant groupings	The detailed clinical characteristics including ethnicity were obtained from patients' health records and previously published in supplemental table 1 in (Jia et al., 2021). We did not consider ethnicity in our current analysis. Other socially relevant information was not included in our current study.
Population characteristics	Multiple myeloma patients (n=7; 5 male, 2 female) were 53 years of age on average with a range of 36 to 67 years. All were newly diagnosed except for 1 relapsed case. All patient characteristics are located in supplementary table 1 of Jia et al. 2021.
Recruitment	Samples were obtained as part of development of a comprehensive tissue and gene registry for hematological malignancies from National University Health System (NUHS) that was approved by the institutional review board (NHG DSRB: 2007/00173). Patients provided written informed consent that permitted the use of biological material in accordance with a protocol that was approved by the institutional review board. Bone marrow (BM) biopsies were obtained at the initial presentation of MM (newly diagnosed), and at the time of relapse (Relapsed MM) as part of routine hematopathology evaluation.
Ethics oversight	Written, informed consent and ethical approval by the National Healthcare Group Domain Specific Review Board (NHG DSRB: 2007/00173) was obtained for human samples, in accordance with the Declaration of Helsinki.

Note that full information on the approval of the study protocol must also be provided in the manuscript.

Field-specific reporting

Please select the one below that is the best fit for your research. If you are not sure, read the appropriate sections before making your selection.

☒ Life sciences ☐ Behavioural & social sciences ☐ Ecological, evolutionary & environmental sciences

For a reference copy of the document with all sections, see nature.com/documents/nr-reporting-summary-flat.pdf

Life sciences study design

All studies must disclose on these points even when the disclosure is negative.

Sample size	No sample-size calculation was performed. Sample sizes followed prevalent guidelines for balancing statistical power/cost and expert advice.
Data exclusions	No data were excluded.
Replication	Endogenous ChIP-seq experiments:<br>Reproducible p52 binding, H3K4 methylation and H3K27 acetylation sites were consistently identified through independent ChIP-seq experiments (n≥2).<br><br>p52 knock-down NGS experiments:<br>Reproducible LP1 H3K27 acetylation changes were consistently measured by H3K27ac ChIP-seq across 3 biological replicates per condition. Reproducible genome wide epigenomic changes for KMS-11 were consistently measured and corroborated across 4 orthogonal approaches (ChIP-seq, ATAC-seq, RNA-seq and Hi-ChIP) using a total of 10 biological replicates per condition. Reproducible genome wide epigenomic changes for MM1.144 were consistently measured and corroborated across 3 orthogonal approaches (ChIP-seq, ATAC-seq and RNA-seq) using a total of 6 biological replicates per condition. Efficiency of p52 knock down replicates was assessed prior to sequencing by western blot and H3K27ac ChIP qPCR.<br><br>Mouse experiments:<br>Reproducible in vivo phenotypes were consistently measured across treatment groups consisting of 5 animals.<br><br>Functional assays:<br>Apoptosis assay staining was done with 3 biological replicates per condition. Proliferation assay (cell trace) staining was done with 3 biological replicates per condition. Adhesion assay was done with technical triplicates and 3 biological replicates per condition.<br><br>Western Blots:<br>Blots shown are a representation of n≥2 replicates
Randomization	Randomization was only relevant to the in vivo component of the study, mice were randomly allocated into control and treatment groups. The in vitro component of the study is exploratory in nature, experiments were designed with fixed known conditions to observe reproducible epigenomic patterns. To deduce their clinical importance, the patterns were retrospectively compared to patient data obtained from past studies that did not include randomization due to ethical constraints.
Blinding	Blinding was not necessary throughout the study due to the objective nature of measurements taken.

Behavioural & social sciences study design

All studies must disclose on these points even when the disclosure is negative.

Study description	*Briefly describe the study type including whether data are quantitative, qualitative, or mixed-methods (e.g. qualitative cross-sectional, quantitative experimental, mixed-methods case study).*
Research sample	*State the research sample (e.g. Harvard university undergraduates, villagers in rural India) and provide relevant demographic information (e.g. age, sex) and indicate whether the sample is representative. Provide a rationale for the study sample chosen. For studies involving existing datasets, please describe the dataset and source.*
Sampling strategy	*Describe the sampling procedure (e.g. random, snowball, stratified, convenience). Describe the statistical methods that were used to predetermine sample size OR if no sample-size calculation was performed, describe how sample sizes were chosen and provide a rationale for why these sample sizes are sufficient. For qualitative data, please indicate whether data saturation was considered, and what criteria were used to decide that no further sampling was needed.*
Data collection	*Provide details about the data collection procedure, including the instruments or devices used to record the data (e.g. pen and paper, computer, eye tracker, video or audio equipment) whether anyone was present besides the participant(s) and the researcher, and whether the researcher was blind to experimental condition and/or the study hypothesis during data collection.*
Timing	*Indicate the start and stop dates of data collection. If there is a gap between collection periods, state the dates for each sample cohort.*

Data exclusions	*If no data were excluded from the analyses, state so OR if data were excluded, provide the exact number of exclusions and the rationale behind them, indicating whether exclusion criteria were pre-established.*
Non-participation	*State how many participants dropped out/declined participation and the reason(s) given OR provide response rate OR state that no participants dropped out/declined participation.*
Randomization	*If participants were not allocated into experimental groups, state so OR describe how participants were allocated to groups, and if allocation was not random, describe how covariates were controlled.*

Ecological, evolutionary & environmental sciences study design

All studies must disclose on these points even when the disclosure is negative.

Study description	*Briefly describe the study. For quantitative data include treatment factors and interactions, design structure (e.g. factorial, nested, hierarchical), nature and number of experimental units and replicates.*
Research sample	*Describe the research sample (e.g. a group of tagged Passer domesticus, all Stenocereus thurberi within Organ Pipe Cactus National Monument), and provide a rationale for the sample choice. When relevant, describe the organism taxa, source, sex, age range and any manipulations. State what population the sample is meant to represent when applicable. For studies involving existing datasets, describe the data and its source.*
Sampling strategy	*Note the sampling procedure. Describe the statistical methods that were used to predetermine sample size OR if no sample-size calculation was performed, describe how sample sizes were chosen and provide a rationale for why these sample sizes are sufficient.*
Data collection	*Describe the data collection procedure, including who recorded the data and how.*
Timing and spatial scale	*Indicate the start and stop dates of data collection, noting the frequency and periodicity of sampling and providing a rationale for these choices. If there is a gap between collection periods, state the dates for each sample cohort. Specify the spatial scale from which the data are taken*
Data exclusions	*If no data were excluded from the analyses, state so OR if data were excluded, describe the exclusions and the rationale behind them, indicating whether exclusion criteria were pre-established.*
Reproducibility	*Describe the measures taken to verify the reproducibility of experimental findings. For each experiment, note whether any attempts to repeat the experiment failed OR state that all attempts to repeat the experiment were successful.*
Randomization	*Describe how samples/organisms/participants were allocated into groups. If allocation was not random, describe how covariates were controlled. If this is not relevant to your study, explain why.*
Blinding	*Describe the extent of blinding used during data acquisition and analysis. If blinding was not possible, describe why OR explain why blinding was not relevant to your study.*

Did the study involve field work? ☐ Yes ☐ No

Field work, collection and transport

Field conditions	*Describe the study conditions for field work, providing relevant parameters (e.g. temperature, rainfall).*
Location	*State the location of the sampling or experiment, providing relevant parameters (e.g. latitude and longitude, elevation, water depth).*
Access & import/export	*Describe the efforts you have made to access habitats and to collect and import/export your samples in a responsible manner and in compliance with local, national and international laws, noting any permits that were obtained (give the name of the issuing authority, the date of issue, and any identifying information).*
Disturbance	*Describe any disturbance caused by the study and how it was minimized.*

Reporting for specific materials, systems and methods

We require information from authors about some types of materials, experimental systems and methods used in many studies. Here, indicate whether each material, system or method listed is relevant to your study. If you are not sure if a list item applies to your research, read the appropriate section before selecting a response.

Materials & experimental systems

n/a	Involved in the study
☐	☒ Antibodies
☐	☒ Eukaryotic cell lines
☒	☐ Palaeontology and archaeology
☐	☒ Animals and other organisms
☒	☐ Clinical data
☒	☐ Dual use research of concern
☒	☐ Plants

Methods

n/a	Involved in the study
☐	☒ ChIP-seq
☐	☒ Flow cytometry
☒	☐ MRI-based neuroimaging

Antibodies

Antibodies used

Western blot: NFKB1(13586; CST, 1:1000), NFKB2 (3017; CST,1:1000), RelB (10544; CST,1:1000), p65 (8242; CST,1:1000), NIK (4994; CST,1:1000), TRAF3 (61095; CST,1:1000), RGS1 (ab154973; abcam,1:1000), pJNK (4668S; CST,1:1000), and p-p38 (4511S; CST,1:1000) rabbit monoclonal antibody. GAPDH (sc-32233; Santa Cruz,1:10000), c-Rel (sc-6955; Santa Cruz,1:500), p-IKBa (9246; CST,1:1000),BCL2 (15071;CST,1:1000), p-cdc2 (4539S; CST,1:1000), cyclin D1 (E3P5S; CST,1:1000), JNK (sc-7345; Santa Cruz,,1:500), c-Jun (sc-74543; Santa Cruz,1:500), p-c-Jun (sc-822; Santa Cruz,1:500), p38α (sc-166182; Santa Cruz,1:500), GRAP2 (sc-73652; Santa Cruz,1:1000), cdc2 (sc-53219; Santa Cruz,1:1000), p53 (2524S; CST,1:1000), p-p53 (sc-377567; Santa Cruz,1:500) and Bax (sc-7480; Santa Cruz,1:500) mouse monoclonal antibody. Anti-rabbit (7074S; CST,1:10000) or anti-mouse (sc-516102; Santa Cruz,1:10000 horseradish peroxidase (HRP)-conjugated secondary antibody.

ChIP: NFKB2 antibody (A300-BL7039; Bethyl Laboratories), H3K27ac antibody (07-360; Merck), H3K27me3 (9733S; CST), H3K4me1 (AB8895-1003; Abcam) or IgG Rabbit (P120-101; Bethyl Laboratories).

Validation

NFKB1(13586; CST). Western blot analysis of extracts from various cell lines and rat spleen using NF-κB1 p105/p50 (D4P4D) Rabbit mAb. Reactivity: Human, mouse, Rat. Sensitivity: Endogenous. Source/Isotype: Rabbit IgG.
NFKB2 (3017; CST). Western blot analysis of extracts from HeLa, and COS cells, using NF-kB2 p100/p52 (18D10) Rabbit mAb. Reactivity: Human, monkey. Sensitivity: Endogenous. Source/Isotype: Rabbit IgG.
RelB (10544; CST). Western blot analysis of extracts from various cell lines using RelB (D7D7W) Rabbit mAb (upper) or β-Actin (D6A8) Rabbit mAb #8457 (lower). KARPAS cell line source: Dr. Abraham Karpas at the University of Cambridge. Reactivity: H Human, mouse, Rat. Sensitivity: Endogenous. Source/Isotype: Rabbit IgG.
p65 (8242; CST). Western blot analysis of extracts from various cell lines using NF-κB p65 (D14E12) XP® Rabbit mAb. Reactivity: Human, mouse, rat, monkey, dog. Sensitivity: Endogenous. Source/Isotype: Rabbit IgG.
NIK (4994; CST). Western blot analysis of extracts from various cell lines, untreated or treated with 10uM MG132, using NIK Antibody #4994. Reactivity: Human, mouse. Sensitivity: Endogenous. Source: Rabbit.
TRAF3 (61095; CST). Western blot anlysis of extracts from various cell lines using TRAF3 (D1N5B) Rabbit mAb (upper), or β-Actin (D6A8) Rabbit mAb (lower). RPMI 8226 cells harbor a heterozygous deletion in the TRAF3 gene resulting in loss of expression. Reactivity: Human. Sensitivity: Endogenous. Source/Isotype: Rabbit IgG.
RGS1 (ab154973; abcam). Anti-RGS1 antibody (ab154973) at 1/1000 dilution + K562 whole cell lysate at 30 µg. Reacts with: Human. Suitable for: WB, IHC-P. Isotype: IgG
pJNK (4668S; CST). Western blot analysis of extracts from 293 cells, untreated or UV-treated, NIH/3T3 cells, untreated or UV-treated and C6 cells, untreated or anisomycin-treated, using Phospho-SAPK/JNK (Thr183/Tyr185) (81E11) Rabbit mAb. Reactivity: Human, mouse, rat, D. melanogaster, S. cerevisiae. Sensitivity: Endogenous. Source/Isotype: Rabbit IgG.
p-p38 (4511S; CST) Western blot analysis of extracts from COS and 293 cells, untreated or UV-treated, using Phospho-p38 MAPK (Thr180/Tyr182) (D3F9) XP® Rabbit mAb (upper) or p38 MAPK Antibody #9212 (lower). Reactivity: Human, mouse, rat, monkey, mink, pig, S. cerevisiae Sensitivity: Endogenous. Source/Isotype: Rabbit IgG.
GAPDH (sc-32233; Santa Cruz). GAPDH (6C5) is recommended for detection of GAPDH of mouse, rat, human, rabbit and Xenopus laevis origin by Western Blotting (starting dilution 1:200, dilution range 1:100-1:1000). Positive Controls: c4 whole cell lysate: sc-364186, Jurkat whole cell lysate: sc-2204 or MOLT-4 cell lysate: sc-2233. GAPDH (6C5) is a mouse monoclonal antibody raised against GAPDH purified from muscle of rabbit origin.
c-Rel (sc-6955; Santa Cruz). c-Rel (B-6) is recommended for detection of c-Rel p75 of mouse, rat and human origin by Western Blotting (starting dilution 1:200, dilution range 1:100-1:1000). C-Rel (B-6) is a mouse monoclonal antibody raised against amino acids 1-300 mapping at6 the N-terminus of c-Rel of human origin.
p-IKBa (9246; CST). Western blot analysis of extracts from NIH/3T3 cells, untreated or TNF-α-treated (#8902, 20 ng/ml) for 5 minutes, using Phospho-IκBα (Ser32/36) (5A5) Mouse mAb #9246 (upper) or IκBα (L35A5) Mouse mAb (Amino-terminal Antigen) #4814, and β-Actin (D6A8) Rabbit mAb #8457 (lower). Reactivity: Human, mouse, rabbit, monkey. Sensitivity: Endogenous. Source/Isotype: Mouse IgG1.
BCL2 (15071, CST). Western blot analysis of extracts from control HeLa cells (lane 1) or Bcl-2 knockout HeLa cells (lane 2) using Bcl-2 (124) Mouse mAb #15071 (upper), or β-actin (13E5) Rabbit mAb #4970 (lower). The absence of signal in the Bcl-2-knockout HeLa cells confirms specificity of the antibody for Bcl-2. Reactivity: Human. Sensitivity: Endogenous. Source/Isotype: Mouse IgG1.
JNK (sc-7345; Santa Cruz). JNK (D-2) is recommended for detection of all JNK1, JNK2 and JNK3 p46 and p54 isoforms of mouse, rat and human origin by Western Blotting (starting dilution 1:200, dilution range 1:100-1:1000). Positive Controls: HeLa whole cell lysate: sc-2200, NIH/3T3 whole cell lysate: sc-2210 or K-562 whole cell lysate: sc-2203. Mouse monoclonal antibody raised against amino acids 1-424 representing full length JNK2 p54 of human origin.
c-Jun (sc-74543; Santa Cruz). c-Jun (G-4) is recommended for detection of c-Jun p39 of mouse, rat and human origin by Western Blotting (starting dilution 1:100, dilution range 1:100-1:1000). Positive Controls: BYDP whole cell lysate: sc-364368, NIH/3T3 whole cell lysate: sc-2210 or NIH/3T3 + PMA nuclear extract: sc-2125. Mouse monoclonal antibody raised against amino acids 1-79 of c-Jun of human origin.
p-c-Jun (sc-822; Santa Cruz). p-c-Jun (KM-1) is recommended for detection of c-Jun p39 phosphorylated on Serine 63 of mouse, rat and human origin by Western Blotting (starting dilution 1:200, dilution range 1:100-1:1000). Positive Controls: A-431 nuclear extract:

sc-2122, NIH/3T3 whole cell lysate: sc-2210 or NIH/3T3 + anisomycin cell lysate: sc-2247. Mouse monoclonal antibody raised against amino acids 56-69 of human c-Jun.
p38α (sc-166182; Santa Cruz). p-p38 MAPK (E-1) is recommended for detection of Tyr 182 phosphorylated p38a MAPK14, p38b MAPK11 and p38g MAPK12 of mouse, rat and human origin by Western Blotting (starting dilution 1:100, dilution range 1:1001:1000). Mouse monoclonal antibody raised against phosphorylated Tyr 182 of p38 a MAPK14 of human origin.
p53 (2524S; CST). Western blot analysis of cellular extracts using p53 (1C12) Mouse mAb. Reactivity: Human, mouse, rabbit, hamster, monkey. Sensitivity: Endogenous. Source/Isotype: Mouse IgG1.
p-p53 (sc-377567; Santa Cruz). p-p53 (D-9) is recommended for detection of Thr 155 phosphorylated p53 of human origin by Western Blotting (starting dilution 1:100, dilution range 1:100-1:1000). Mouse monoclonal antibody specific for an epitope mapping between amino acids 150-163 Thr 155 of p53 of human origin.
Bax (sc-7480; Santa Cruz). Bax (B-9) is recommended for detection of Baxα and Baxβ of mouse, rat and human origin by Western Blotting (starting dilution 1:200, dilution range 1:100-1:1000). Positive Controls: Raji whole cell lysate: sc-364236, NAMALWA cell lysate: sc-2234 or Jurkat whole cell lysate: sc-2204. Mouse monoclonal antibody raised against amino acids 1-171 of Baxα of mouse origin.
p-cdc2 (4539S; CST). Phospho-cdc2 (Tyr15) is recommended for detection endogenous levels of cdc2 protein only when phosphorylated at tyrosine 15. Souce/isotype: Rabbit. Species reactivity: Human, mouse, rabbit, monkey. Application: western blotting (1:1000 dilution). Source: Monoclonal antibody and produced by immunizing animals with a synthetic phosphopeptide corresponding to residues surrounding Tyr15 of human cdc2. Western blot analysis of extracts from C6 cells and HeLa cells.
Cyclin D1 (E3P5S;CST): Cyclin D1 (E3P5S) XP® Rabbit mAb recommended for detection of endogenous levels of total cyclin D1 protein. Source/isotype: Rabbit IgG. Species reactivity: Human, mouse, rat. Application: western blotting (1:1000 dilution). Souce: Monoclonal antibody is produced by immunizing animals with a synthetic peptide corresponding to residues surrounding Ala284 of human cyclin D1 protein. Western blot analysis of extracts from SH-SY5Y, BJ, U266B1,K-562 cell lines.
GRAP2 (sc-73652): Gads Antibody (UW40) is recommended for detection of Gads of mouse, rat and human origin by WB, IP, IF and ELISA. Source/isotype: Gads (UW40) is a mouse monoclonal antibody raised against GST-fusion protein expressing Gads of human origin. Species reactivity: Human, mouse , rat. Application: western blotting (1:100-1:1000 range). Positive controls: Western blot analysis with cell lysate of Jurkat, HeLa and MOLT-4 cells.
cdc2 (sc-53219): Cdk1/Cdk2/cdc2 Antibody (AN21.2) is recommended for detection of Cdk1/Cdk2/cdc2 of mouse, rat and human origin by WB, IP, IF and IHC(P). Souce/isotype: Cdk1/Cdk2/cdc2 (AN21.2) is a mouse monoclonal antibody raised against recombinant Cdc2 of human origin. Species reactivity: Human, mouse and rat. Application: western blotting (1:100-1:1000 range). Positive controls: western blot analysis with whole cell lysate of K-562, HeLa and NAMALWA cells.
sc-2200 or NAMALWA cell lysate: sc-2234.

NFKB2 antibody (A300-BL7039; Bethyl Laboratories)
H3K27ac antibody (07-360; Merck). Anti-acetyl-Histone H3 (Lys27) Antibody is a rabbit polyclonal antibody for detection of Histone H3 acetylated on lysine 27. Also known as Anti-H3K27ac this antibody is published in peer reviewed journals and is specificity verified by dot blot (DB) and validated in ChIP, ChIP-seq. Reactivity: Human, Vertebrates, Yeast. Host: rabbit polyclonal antibody IgG.

H3K27me3 (9733S; CST). Tri-Methyl-Histone H3 (Lys27) (C36B11) Rabbit mAb detects endogenous levels of histone H3 only when tri-methylated on Lys27. The antibody does not cross-react with non-methylated, mono-methylated or di-methylated Lys27. In addition, the antibody does not cross-react with mono-methylated, di-methylated or tri-methylated histone H3 at Lys4, Lys9, Lys36 or Histone H4 at Lys20. Has been validated using SimpleChIP® Enzymatic Chromatin IP Kits. Reactivity: Human, Mouse, Rat, Monkey. Host: rabbit monoclonal antibody IgG.

H3K4me1 (AB8895-1003; Abcam). Specific for mono-methylated Lysine 4 of histone H3. Does not recognise di- or tri-methyl Lysine 4 nor methylation at Lysine 9. Validated for ChIP in Human cell line U-2 OS cells using Abcam X-ChIP protocol. Host: rabbit polyclonal antibody IgG.

IgG Rabbit (P120-101; Bethyl Laboratories). By immunoelectrophoresis the IgG was shown to 1) react with antiserum specific for rabbit IgG, 2) not react with antiserum specific for IgA or IgM, and 3) produce a single precipitin arc with antiserum against rabbit serum identical to that produced with anti-IgG antisera.

Eukaryotic cell lines

Policy information about cell lines and Sex and Gender in Research

Cell line source(s)	MM1.144 (female), L363 (female), ANBL6 (female), U266 (male), KMS-11 (female), RPMI8226 (male), JJN3 (female), XG7 (female) and H929 (female) were kindly provided by Prof. Leif Bergsagel (Mayo Clinic, Scottsdale, AZ, USA). The MM1.S (female) cell line was obtained from ATCC while LP1 (female) and MOLP8 (male) were obtained from the German Collection of Microorganisms and Cell Cultures. Patient derived CD138 positive plasma cells were obtained from 5 male and 2 female patients. Informed consent and ethical approval by institutional review boards (IRB) were obtained (DSRB: 2007/00173), in accordance with the Declaration of Helsinki.
Authentication	Cell lines were authenticated using STR (Applied Biosystems).
Mycoplasma contamination	Cell lines were tested to be negative for Mycoplasma using Mycoplasma PCR Detection Kit (Abm, G238).
Commonly misidentified lines (See ICLAC register)	According to the ICLAC, H929 (NCI-H929) has been reported to be contaminated with K-562 however authentic stock is known to exist. Our stocks were recently authenticated by STR analysis (Centre for Translational Research and Diagnostics, National University of Singapore).

Palaeontology and Archaeology

Specimen provenance	*Provide provenance information for specimens and describe permits that were obtained for the work (including the name of the issuing authority, the date of issue, and any identifying information). Permits should encompass collection and, where applicable, export.*
Specimen deposition	*Indicate where the specimens have been deposited to permit free access by other researchers.*
Dating methods	*If new dates are provided, describe how they were obtained (e.g. collection, storage, sample pretreatment and measurement), where they were obtained (i.e. lab name), the calibration program and the protocol for quality assurance OR state that no new dates are provided.*

☐ Tick this box to confirm that the raw and calibrated dates are available in the paper or in Supplementary Information.

Ethics oversight	*Identify the organization(s) that approved or provided guidance on the study protocol, OR state that no ethical approval or guidance was required and explain why not.*

Note that full information on the approval of the study protocol must also be provided in the manuscript.

Animals and other research organisms

Policy information about studies involving animals; ARRIVE guidelines recommended for reporting animal research, and Sex and Gender in Research

Laboratory animals	Orthotopic experiments: NOD-scid IL2rγnull (NSG) mice were used at 6 weeks post-partum.<br>Xenograft experiments: Balb/c RAG -/- IL2Rγ -/- mice (6-10 weeks) used.
Wild animals	No wild animals were used in the study.
Reporting on sex	Orthotopic experiments: 15 male mice were used but sex was not considered in the study design.<br>Xenograft experiments: Six male and nine female mice were used. Each treatment condition had a mixture of male and female mice.
Field-collected samples	No field-collected samples were used in the study.
Ethics oversight	Xenograft experiments: Animal studies were approved by Institutional Animal Care and Use Committee of Nanyang Technological University Singapore (NTU-ARF; AUP: A21070)<br>Orthotopic experiments: Agency for Science, Technology and Research (A*STAR) - Institutional Animal Care and Use Committee (IACUC) approved the study protocol (#221738).

Note that full information on the approval of the study protocol must also be provided in the manuscript.

Clinical data

Policy information about clinical studies
All manuscripts should comply with the ICMJE guidelines for publication of clinical research and a completed CONSORT checklist must be included with all submissions.

Clinical trial registration	*Provide the trial registration number from ClinicalTrials.gov or an equivalent agency.*
Study protocol	*Note where the full trial protocol can be accessed OR if not available, explain why.*
Data collection	*Describe the settings and locales of data collection, noting the time periods of recruitment and data collection.*
Outcomes	*Describe how you pre-defined primary and secondary outcome measures and how you assessed these measures.*

Dual use research of concern

Policy information about dual use research of concern

Hazards

Could the accidental, deliberate or reckless misuse of agents or technologies generated in the work, or the application of information presented in the manuscript, pose a threat to:

No | Yes

- [ ] [ ] Public health
- [ ] [ ] National security
- [ ] [ ] Crops and/or livestock
- [ ] [ ] Ecosystems
- [ ] [ ] Any other significant area

Experiments of concern

Does the work involve any of these experiments of concern:

No | Yes

- [ ] [ ] Demonstrate how to render a vaccine ineffective
- [ ] [ ] Confer resistance to therapeutically useful antibiotics or antiviral agents
- [ ] [ ] Enhance the virulence of a pathogen or render a nonpathogen virulent
- [ ] [ ] Increase transmissibility of a pathogen
- [ ] [ ] Alter the host range of a pathogen
- [ ] [ ] Enable evasion of diagnostic/detection modalities
- [ ] [ ] Enable the weaponization of a biological agent or toxin
- [ ] [ ] Any other potentially harmful combination of experiments and agents

Plants

Seed stocks	*Report on the source of all seed stocks or other plant material used. If applicable, state the seed stock centre and catalogue number. If plant specimens were collected from the field, describe the collection location, date and sampling procedures.*
Novel plant genotypes	*Describe the methods by which all novel plant genotypes were produced. This includes those generated by transgenic approaches, gene editing, chemical/radiation-based mutagenesis and hybridization. For transgenic lines, describe the transformation method, the number of independent lines analyzed and the generation upon which experiments were performed. For gene-edited lines, describe the editor used, the endogenous sequence targeted for editing, the targeting guide RNA sequence (if applicable) and how the editor was applied.*
Authentication	*Describe any authentication procedures for each seed stock used or novel genotype generated. Describe any experiments used to assess the effect of a mutation and, where applicable, how potential secondary effects (e.g. second site T-DNA insertions, mosiacism, off-target gene editing) were examined.*

ChIP-seq

Data deposition

- [x] Confirm that both raw and final processed data have been deposited in a public database such as GEO.
- [x] Confirm that you have deposited or provided access to graph files (e.g. BED files) for the called peaks.

Data access links
May remain private before publication.

GSE230526

Files in database submission

H3K27ac_MM1144_rep1.narrowPeak.gz
H3K27ac_MM1144_rep2.narrowPeak.gz
H3K27me3_KMS11_rep1.narrowPeak.gz
H3K27me3_KMS11_rep2.narrowPeak.gz
H3K4me1_KMS11_rep1.narrowPeak.gz
H3K4me1_KMS11_rep2.narrowPeak.gz
p52KD_H3K27ac_MM1144_ctrl_rep1.narrowPeak.gz
p52KD_H3K27ac_MM1144_ctrl_rep2.narrowPeak.gz
p52KD_H3K27ac_MM1144_trgt_rep1.narrowPeak.gz
p52KD_H3K27ac_MM1144_trgt_rep2.narrowPeak.gz
p52_MM1144_rep1.narrowPeak.gz
p52_MM1144_rep2.narrowPeak.gz
H3K27ac_JJN3_rep1.narrowPeak
H3K27ac_JJN3_rep2.narrowPeak
H3K27ac_JJN3_rep3.narrowPeak
H3K27ac_KMS11_rep1.narrowPeak
H3K27ac_KMS11_rep2.narrowPeak
H3K27ac_KMS11_rep3.narrowPeak
H3K27ac_LP1_rep1.narrowPeak

H3K27ac_LP1_rep2.narrowPeak
H3K27ac_LP1_rep3.narrowPeak
H3K27ac_MM1S_rep1.narrowPeak
H3K27ac_MM1S_rep2.narrowPeak
H3K27ac_MM1S_rep3.narrowPeak
H3K27ac_U266_rep1.narrowPeak
H3K27ac_U266_rep2.narrowPeak
H3K27ac_U266_rep3.narrowPeak
p52_JJN3_rep1.narrowPeak
p52_JJN3_rep2.narrowPeak
p52_JJN3_rep3.narrowPeak
p52_KMS11_rep1.narrowPeak
p52_KMS11_rep2.narrowPeak
p52_KMS11_rep3.narrowPeak
p52_KMS11_rep4.narrowPeak
p52_LP1_rep1.narrowPeak
p52_LP1_rep2.narrowPeak
p52_LP1_rep3.narrowPeak
p52_MM1S_rep1.narrowPeak
p52_MM1S_rep2.narrowPeak
p52_MM1S_rep3.narrowPeak
p52_U266_rep1.narrowPeak
p52_U266_rep2.narrowPeak
p52_U266_rep3.narrowPeak
p52KD_H3K27ac_KMS11_ctrl_rep1.narrowPeak
p52KD_H3K27ac_KMS11_ctrl_rep2.narrowPeak
p52KD_H3K27ac_KMS11_ctrl_rep3.narrowPeak
p52KD_H3K27ac_KMS11_trgt_rep1.narrowPeak
p52KD_H3K27ac_KMS11_trgt_rep2.narrowPeak
p52KD_H3K27ac_KMS11_trgt_rep3.narrowPeak
p52KD_H3K27ac_LP1_ctrl_rep1.narrowPeak.gz
p52KD_H3K27ac_LP1_ctrl_rep2.narrowPeak.gz
p52KD_H3K27ac_LP1_ctrl_rep3.narrowPeak.gz
p52KD_H3K27ac_LP1_trgt_rep1.narrowPeak.gz
p52KD_H3K27ac_LP1_trgt_rep2.narrowPeak.gz
p52KD_H3K27ac_LP1_trgt_rep3.narrowPeak.gz
H3K27ac_MM1144_rep1_1.fastq.gz
H3K27ac_MM1144_rep1_2.fastq.gz
H3K27ac_MM1144_rep2_1.fastq.gz
H3K27ac_MM1144_rep2_2.fastq.gz
H3K27me3_KMS11_rep1_1.fastq.gz
H3K27me3_KMS11_rep1_2.fastq.gz
H3K27me3_KMS11_rep2_1.fastq.gz
H3K27me3_KMS11_rep2_2.fastq.gz
H3K4me1_KMS11_rep1_1.fastq.gz
H3K4me1_KMS11_rep1_2.fastq.gz
H3K4me1_KMS11_rep2_1.fastq.gz
H3K4me1_KMS11_rep2_2.fastq.gz
p52KD_H3K27ac_MM1144_ctrl_rep1_run1_1.fastq.gz
p52KD_H3K27ac_MM1144_ctrl_rep1_run1_2.fastq.gz
p52KD_H3K27ac_MM1144_ctrl_rep1_run2_1.fastq.gz
p52KD_H3K27ac_MM1144_ctrl_rep1_run2_2.fastq.gz
p52KD_H3K27ac_MM1144_ctrl_rep2_run1_1.fastq.gz
p52KD_H3K27ac_MM1144_ctrl_rep2_run1_2.fastq.gz
p52KD_H3K27ac_MM1144_ctrl_rep2_run2_1.fastq.gz
p52KD_H3K27ac_MM1144_ctrl_rep2_run2_2.fastq.gz
p52KD_H3K27ac_MM1144_trgt_rep1_run1_1.fastq.gz
p52KD_H3K27ac_MM1144_trgt_rep1_run1_2.fastq.gz
p52KD_H3K27ac_MM1144_trgt_rep1_run2_1.fastq.gz
p52KD_H3K27ac_MM1144_trgt_rep1_run2_2.fastq.gz
p52KD_H3K27ac_MM1144_trgt_rep2_run1_1.fastq.gz
p52KD_H3K27ac_MM1144_trgt_rep2_run1_2.fastq.gz
p52KD_H3K27ac_MM1144_trgt_rep2_run2_1.fastq.gz
p52KD_H3K27ac_MM1144_trgt_rep2_run2_2.fastq.gz
p52_MM1144_rep1_1.fastq.gz
p52_MM1144_rep1_2.fastq.gz
p52_MM1144_rep2_1.fastq.gz
p52_MM1144_rep2_2.fastq.gz
H3K27ac_JJN3_rep1_1.fastq.gz
H3K27ac_JJN3_rep1_2.fastq.gz
H3K27ac_JJN3_rep2_1.fastq.gz
H3K27ac_JJN3_rep2_2.fastq.gz
H3K27ac_JJN3_rep3_1.fastq.gz
H3K27ac_JJN3_rep3_2.fastq.gz
H3K27ac_KMS11_rep1.fastq.gz
H3K27ac_KMS11_rep2.fastq.gz

H3K27ac_KMS11_rep3.fastq.gz
H3K27ac_LP1_rep1_1.fastq.gz
H3K27ac_LP1_rep1_2.fastq.gz
H3K27ac_LP1_rep2_1.fastq.gz
H3K27ac_LP1_rep2_2.fastq.gz
H3K27ac_LP1_rep3_1.fastq.gz
H3K27ac_LP1_rep3_2.fastq.gz
H3K27ac_MM1S_rep1.fastq.gz
H3K27ac_MM1S_rep2.fastq.gz
H3K27ac_MM1S_rep3.fastq.gz
H3K27ac_U266_rep1.fastq.gz
H3K27ac_U266_rep2.fastq.gz
H3K27ac_U266_rep3.fastq.gz
input_JJN3_rep1_1.fastq.gz
input_JJN3_rep1_2.fastq.gz
input_KMS11_rep1.fastq.gz
input_KMS11_ctrl_rep1_1.fastq.gz
input_KMS11_ctrl_rep1_2.fastq.gz
input_LP1_rep1_1.fastq.gz
input_LP1_rep1_2.fastq.gz
input_MM1S_rep1.fastq.gz
input_U266_rep1.fastq.gz
p52_JJN3_rep1_1.fastq.gz
p52_JJN3_rep1_2.fastq.gz
p52_JJN3_rep2_1.fastq.gz
p52_JJN3_rep2_2.fastq.gz
p52_JJN3_rep3_1.fastq.gz
p52_JJN3_rep3_2.fastq.gz
p52_KMS11_rep1.fastq.gz
p52_KMS11_rep2.fastq.gz
p52_KMS11_rep3.fastq.gz
p52_KMS11_rep4_1.fastq.gz
p52_KMS11_rep4_2.fastq.gz
p52_LP1_rep1_1.fastq.gz
p52_LP1_rep1_2.fastq.gz
p52_LP1_rep2_1.fastq.gz
p52_LP1_rep2_2.fastq.gz
p52_LP1_rep3_1.fastq.gz
p52_LP1_rep3_2.fastq.gz
p52_MM1S_rep1.fastq.gz
p52_MM1S_rep2.fastq.gz
p52_MM1S_rep3.fastq.gz
p52_U266_rep1.fastq.gz
p52_U266_rep2.fastq.gz
p52_U266_rep3.fastq.gz
p52KD_H3K27ac_KMS11_ctrl_rep1_1.fastq.gz
p52KD_H3K27ac_KMS11_ctrl_rep1_2.fastq.gz
p52KD_H3K27ac_KMS11_ctrl_rep2_1.fastq.gz
p52KD_H3K27ac_KMS11_ctrl_rep2_2.fastq.gz
p52KD_H3K27ac_KMS11_ctrl_rep3_1.fastq.gz
p52KD_H3K27ac_KMS11_ctrl_rep3_2.fastq.gz
p52KD_H3K27ac_KMS11_trgt_rep1_1.fastq.gz
p52KD_H3K27ac_KMS11_trgt_rep1_2.fastq.gz
p52KD_H3K27ac_KMS11_trgt_rep2_1.fastq.gz
p52KD_H3K27ac_KMS11_trgt_rep2_2.fastq.gz
p52KD_H3K27ac_KMS11_trgt_rep3_1.fastq.gz
p52KD_H3K27ac_KMS11_trgt_rep3_2.fastq.gz
p52KD_H3K27ac_LP1_ctrl_rep1_1.fastq.gz
p52KD_H3K27ac_LP1_ctrl_rep1_2.fastq.gz
p52KD_H3K27ac_LP1_ctrl_rep2_1.fastq.gz
p52KD_H3K27ac_LP1_ctrl_rep2_2.fastq.gz
p52KD_H3K27ac_LP1_ctrl_rep3_1.fastq.gz
p52KD_H3K27ac_LP1_ctrl_rep3_2.fastq.gz
p52KD_H3K27ac_LP1_trgt_rep1_1.fastq.gz
p52KD_H3K27ac_LP1_trgt_rep1_2.fastq.gz
p52KD_H3K27ac_LP1_trgt_rep2_1.fastq.gz
p52KD_H3K27ac_LP1_trgt_rep2_2.fastq.gz
p52KD_H3K27ac_LP1_trgt_rep3_1.fastq.gz
p52KD_H3K27ac_LP1_trgt_rep3_2.fastq.gz
H3K27ac_MM1144_rep1.bw
H3K27ac_MM1144_rep2.bw
H3K27me3_KMS11_rep1.bw
H3K27me3_KMS11_rep2.bw
H3K4me1_KMS11_rep1.bw
H3K4me1_KMS11_rep2.bw
p52KD_H3K27ac_MM1144_ctrl_rep1.bw

p52KD_H3K27ac_MM1144_ctrl_rep2.bw
p52KD_H3K27ac_MM1144_trgt_rep1.bw
p52KD_H3K27ac_MM1144_trgt_rep2.bw
p52_MM1144_rep1.bw
p52_MM1144_rep2.bw
H3K27ac_JJN3_rep1.bw
H3K27ac_JJN3_rep2.bw
H3K27ac_JJN3_rep3.bw
H3K27ac_KMS11_rep1.bw
H3K27ac_KMS11_rep2.bw
H3K27ac_KMS11_rep3.bw
H3K27ac_LP1_rep1.bw
H3K27ac_LP1_rep2.bw
H3K27ac_LP1_rep3.bw
H3K27ac_MM1S_rep1.bw
H3K27ac_MM1S_rep2.bw
H3K27ac_MM1S_rep3.bw
H3K27ac_U266_rep1.bw
H3K27ac_U266_rep2.bw
H3K27ac_U266_rep3.bw
input_JJN3_rep1.bw
input_KMS11_rep1.bw
input_KMS11_ctrl_rep1.bw
input_LP1_rep1.bw
input_MM1S_rep1.bw
input_U266_rep1.bw
p52_JJN3_rep1.bw
p52_JJN3_rep2.bw
p52_JJN3_rep3.bw
p52_KMS11_rep1.bw
p52_KMS11_rep2.bw
p52_KMS11_rep3.bw
p52_KMS11_rep4.bw
p52_LP1_rep1.bw
p52_LP1_rep2.bw
p52_LP1_rep3.bw
p52_MM1S_rep1.bw
p52_MM1S_rep2.bw
p52_MM1S_rep3.bw
p52_U266_rep1.bw
p52_U266_rep2.bw
p52_U266_rep3.bw
p52KD_H3K27ac_KMS11_ctrl_rep1.bw
p52KD_H3K27ac_KMS11_ctrl_rep2.bw
p52KD_H3K27ac_KMS11_ctrl_rep3.bw
p52KD_H3K27ac_KMS11_trgt_rep1.bw
p52KD_H3K27ac_KMS11_trgt_rep2.bw
p52KD_H3K27ac_KMS11_trgt_rep3.bw
p52KD_H3K27ac_LP1_ctrl_rep1.bw
p52KD_H3K27ac_LP1_ctrl_rep2.bw
p52KD_H3K27ac_LP1_ctrl_rep3.bw
p52KD_H3K27ac_LP1_trgt_rep1.bw
p52KD_H3K27ac_LP1_trgt_rep2.bw
p52KD_H3K27ac_LP1_trgt_rep3.bw

Genome browser session (e.g. UCSC)

TBC

Methodology

Replicates

Biological replication n≥2

Sequencing depth

id	read depth	type	platform	length
JJN3_CHIP_inp_rep1	28281263	paired-end	Illumina HiSeq X	150 bp
JJN3_CHIP_k27_rep1	41042729	paired-end	Illumina HiSeq X	150 bp
JJN3_CHIP_k27_rep2	39108021	paired-end	Illumina HiSeq X	150 bp
JJN3_CHIP_k27_rep3	42294496	paired-end	Illumina HiSeq X	150 bp
JJN3_CHIP_p52_rep1	55712485	paired-end	Illumina HiSeq X	150 bp
JJN3_CHIP_p52_rep2	45924424	paired-end	Illumina HiSeq X	150 bp
JJN3_CHIP_p52_rep3	34072149	paired-end	Illumina HiSeq X	150 bp
KMS11_CHIP_ctrl_inp_rep1	36610586	paired-end	Illumina HiSeq X	150 bp
KMS11_CHIP_ctrl_k27_rep1	70223680	paired-end	Illumina HiSeq X	150 bp
KMS11_CHIP_ctrl_k27_rep2	62389837	paired-end	Illumina HiSeq X	150 bp
KMS11_CHIP_ctrl_k27_rep3	47709583	paired-end	Illumina HiSeq X	150 bp

KMS11_CHIP_inp_rep1	61687103	single	Illumina NextSeq 500	150 bp
KMS11_CHIP_k27_rep1	26995737	single	Illumina NextSeq 500	150 bp
KMS11_CHIP_k27_rep2	70167862	single	Illumina NextSeq 500	150 bp
KMS11_CHIP_k27_rep3	88650549	single	Illumina NextSeq 500	150 bp
KMS11_CHIP_p52_rep1	77951605	single	Illumina NextSeq 500	150 bp
KMS11_CHIP_p52_rep2	102153487	single	Illumina NextSeq 500	150 bp
KMS11_CHIP_p52_rep3	71508768	single	Illumina NextSeq 500	150 bp
KMS11_CHIP_p52_rep4	32764998	paired-end	Illumina HiSeq X	150 bp
KMS11_CHIP_trgt_k27_rep1	46208922	paired-end	Illumina HiSeq X	150 bp
KMS11_CHIP_trgt_k27_rep2	33132176	paired-end	Illumina HiSeq X	150 bp
KMS11_CHIP_trgt_k27_rep3	55658988	paired-end	Illumina HiSeq X	150 bp
LP1_CHIP_ctrl_k27_rep1	39044951	paired-end	Illumina HiSeq X	150 bp
LP1_CHIP_ctrl_k27_rep2	36170130	paired-end	Illumina HiSeq X	150 bp
LP1_CHIP_ctrl_k27_rep3	36094249	paired-end	Illumina HiSeq X	150 bp
LP1_CHIP_inp_rep1	22666930	paired-end	Illumina HiSeq X	150 bp
LP1_CHIP_k27_rep1	31217660	paired-end	Illumina HiSeq X	150 bp
LP1_CHIP_k27_rep2	35501888	paired-end	Illumina HiSeq X	150 bp
LP1_CHIP_k27_rep3	37641298	paired-end	Illumina HiSeq X	150 bp
LP1_CHIP_p52_rep1	42042698	paired-end	Illumina HiSeq X	150 bp
LP1_CHIP_p52_rep2	51868327	paired-end	Illumina HiSeq X	150 bp
LP1_CHIP_p52_rep3	45541181	paired-end	Illumina HiSeq X	150 bp
LP1_CHIP_trgt_k27_rep1	34605005	paired-end	Illumina HiSeq X	150 bp
LP1_CHIP_trgt_k27_rep2	43051372	paired-end	Illumina HiSeq X	150 bp
LP1_CHIP_trgt_k27_rep3	40762718	paired-end	Illumina HiSeq X	150 bp
MM1S_CHIP_inp_rep1	75075349	single	Illumina NextSeq 500	150 bp
MM1S_CHIP_k27_rep1	56821878	single	Illumina NextSeq 500	150 bp
MM1S_CHIP_k27_rep2	72814947	single	Illumina NextSeq 500	150 bp
MM1S_CHIP_k27_rep3	88778833	single	Illumina NextSeq 500	150 bp
MM1S_CHIP_p52_rep1	42519604	single	Illumina NextSeq 500	150 bp
MM1S_CHIP_p52_rep2	65764873	single	Illumina NextSeq 500	150 bp
MM1S_CHIP_p52_rep3	104249710	single	Illumina NextSeq 500	150 bp
U266_CHIP_inp_rep1	77235151	single	Illumina NextSeq 500	150 bp
U266_CHIP_k27_rep1	44856331	single	Illumina NextSeq 500	150 bp
U266_CHIP_k27_rep2	55972996	single	Illumina NextSeq 500	150 bp
U266_CHIP_k27_rep3	84565564	single	Illumina NextSeq 500	150 bp
U266_CHIP_p52_rep1	32307618	single	Illumina NextSeq 500	150 bp
U266_CHIP_p52_rep2	69104441	single	Illumina NextSeq 500	150 bp
U266_CHIP_p52_rep3	47234981	single	Illumina NextSeq 500	150 bp
KMS11_CHIP_K27me3_rep1	28420815	paired-end	Illumina HiSeq X	150 bp
KMS11_CHIP_K27me3_rep2	33815821	paired-end	Illumina HiSeq X	150 bp
KMS11_CHIP_K4me1_rep1	24320363	paired-end	Illumina HiSeq X	150 bp
KMS11_CHIP_K4me1_rep2	27211592	paired-end	Illumina HiSeq X	150 bp
MM1144_CHIP_K27ac_ctrl_rep1	23164598	paired-end	Illumina NovaSeq X Plus	150 bp
MM1144_CHIP_K27ac_ctrl_rep2	24905920	paired-end	Illumina NovaSeq X Plus	150 bp
MM1144_CHIP_K27ac_rep1	50985327	paired-end	Illumina HiSeq X	150 bp
MM1144_CHIP_K27ac_rep2	42000661	paired-end	Illumina HiSeq X	150 bp
MM1144_CHIP_K27ac_trgt_rep1	25232978	paired-end	Illumina NovaSeq X Plus	150 bp
MM1144_CHIP_K27ac_trgt_rep2	24001180	paired-end	Illumina NovaSeq X Plus	150 bp
MM1144_CHIP_p52_rep1	38639426	paired-end	Illumina HiSeq X	150 bp
MM1144_CHIP_p52_rep2	45224275	paired-end	Illumina HiSeq X	150 bp

Antibodies

NFKB2 antibody (A300-BL7039; Bethyl Laboratories), H3K27ac antibody (07-360; Merck), H3K27me3 (9733S; CST), H3K4me1 (AB8895-1003; Abcam) or IgG Rabbit (P120-101; Bethyl Laboratories).

Peak calling parameters

All ChIP-seq (except LP1 p52KD) processed with in-house ChIP-seq pipeline:
bwa mem -t 4 -R "@RG\tID:${ID}\tSM:${ID}" ${HG38}.fa ${REPX_1}.fq ${REPX_2}.fq | samblaster -M -i stdin -o stdout | samtools view -Sb - > ${REPX}.bam
macs2 callpeak -t ${REPX}.bam -g ${GENOMESIZE} –outdir ${REPX_DIR} –name ${REPX} –bdg –SPMR -q 0.05

LP1 and MM1.144 p52KD ChIP-seq processed with ENCODE ChIP-seq pipeline:
bowtie2 -X2000 --mm --threads ${CORES} -x ${BWT2_IDX} -1 ${REPX_1}.fq -2 ${REPX_2}.fq 2 > ${LOG} | samtools view -Su /dev/stdin | samtools sort - > ${REPX}.bam
macs2 callpeak -t ${REPX}.tagAlign.gz -c ${INPUT}.tagAlign.gz -f BED -n ${REPX_DIR_PREFIX} -g ${GENOMESIZE} -p 1e-2 --nomodel --shift 0 --extsize ${FRAGLEN} --keep-dup all -B –SPMR

Data quality

Quality control of ChIP-seq peaks was performed using ChIP-QC. Consistency of replicates were ascertained using IDR and PCA.
Narrow peaks identified using MACS2 using an FDR threshold of 5%:
47318 H3K27ac/JJN3_rep1.narrowPeak
47772 H3K27ac/JJN3_rep2.narrowPeak
49928 H3K27ac/JJN3_rep3.narrowPeak
58421 H3K27ac/KMS11_rep1.narrowPeak
52665 H3K27ac/KMS11_rep2.narrowPeak
42428 H3K27ac/KMS11_rep3.narrowPeak
37439 H3K27ac/LP1_rep1.narrowPeak
42942 H3K27ac/LP1_rep2.narrowPeak
38694 H3K27ac/LP1_rep3.narrowPeak

49838 H3K27ac/MM1S_rep1.narrowPeak
42391 H3K27ac/MM1S_rep2.narrowPeak
42523 H3K27ac/MM1S_rep3.narrowPeak
47318 H3K27ac/U266_rep1.narrowPeak
51904 H3K27ac/U266_rep2.narrowPeak
43842 H3K27ac/U266_rep3.narrowPeak
13734 p52/JJN3_rep1.narrowPeak
8838 p52/JJN3_rep2.narrowPeak
9646 p52/JJN3_rep3.narrowPeak
41042 p52KD_H3K27ac/KMS11_ctrl_rep1.narrowPeak
37931 p52KD_H3K27ac/KMS11_ctrl_rep2.narrowPeak
39997 p52KD_H3K27ac/KMS11_ctrl_rep3.narrowPeak
35241 p52KD_H3K27ac/KMS11_trgt_rep1.narrowPeak
33844 p52KD_H3K27ac/KMS11_trgt_rep2.narrowPeak
40731 p52KD_H3K27ac/KMS11_trgt_rep3.narrowPeak
5644 p52/KMS11_rep1.narrowPeak
2804 p52/KMS11_rep2.narrowPeak
11956 p52/KMS11_rep3.narrowPeak
16630 p52/KMS11_rep4.narrowPeak
4727 p52/LP1_rep1.narrowPeak
2317 p52/LP1_rep2.narrowPeak
3766 p52/LP1_rep3.narrowPeak
14745 p52/MM1S_rep1.narrowPeak
7543 p52/MM1S_rep2.narrowPeak
12238 p52/MM1S_rep3.narrowPeak
3504 p52/U266_rep1.narrowPeak
5801 p52/U266_rep2.narrowPeak
12777 p52/U266_rep3.narrowPeak
Narrow peaks identified using MACS2 using a pval threshold of 1% followed by 5% FDR filtering per condition:
103297 85910 H3K27ac_MM1144_rep1.narrowPeak.gz
157762 110954 H3K27ac_MM1144_rep2.narrowPeak.gz
116688 100795 H3K27me3_KMS11_rep1.narrowPeak.gz
87486 80514 H3K27me3_KMS11_rep2.narrowPeak.gz
168977 155656 H3K4me1_KMS11_rep1.narrowPeak.gz
153034 148133 H3K4me1_KMS11_rep2.narrowPeak.gz
97109 84011 p52KD_H3K27ac_MM1144_ctrl_rep1.narrowPeak.gz
135058 115481 p52KD_H3K27ac_MM1144_ctrl_rep2.narrowPeak.gz
155522 108290 p52KD_H3K27ac_MM1144_trgt_rep1.narrowPeak.gz
159947 117725 p52KD_H3K27ac_MM1144_trgt_rep2.narrowPeak.gz
285007 166762 p52_MM1144_rep1.narrowPeak.gz
179234 133773 p52_MM1144_rep2.narrowPeak.gz
47045 44734 p52KD_H3K27ac_LP1_ctrl_rep1.narrowPeak.gz
46520 43829 p52KD_H3K27ac_LP1_ctrl_rep2.narrowPeak.gz
53842 50607 p52KD_H3K27ac_LP1_ctrl_rep3.narrowPeak.gz
52058 47737 p52KD_H3K27ac_LP1_trgt_rep1.narrowPeak.gz
42482 40473 p52KD_H3K27ac_LP1_trgt_rep2.narrowPeak.gz
66101 60361 p52KD_H3K27ac_LP1_trgt_rep3.narrowPeak.gz

Software

ChIP-seq analyses were performed using bowtie2 or bwa mem for alignments and MACS2 for peak calling.
Differential binding and integration was performed in R with DiffBind or DEseq2.

Flow Cytometry

Plots

Confirm that:

- [ ] The axis labels state the marker and fluorochrome used (e.g. CD4-FITC).
- [ ] The axis scales are clearly visible. Include numbers along axes only for bottom left plot of group (a 'group' is an analysis of identical markers).
- [ ] All plots are contour plots with outliers or pseudocolor plots.
- [ ] A numerical value for number of cells or percentage (with statistics) is provided.

Methodology

Sample preparation

NFKB2 knockdown samples: Inducible system (TLCV2) to express Cas9 and GFP was carried out using doxycycline. Cells with GFP expression sorted and collected. Constitutive Cas9 system (LentiV2) was selected by puromycin. Cells that survived the selection were collected.
Cell proliferation assay (cell trace): Cells labelled with CellTrace™ Blue dye (Invitrogen) for 20 min at 37°C in the dark. Reaction was quenched with complete media for 5 min. Cells were resuspended in fresh media and analysed by FACS.
Cell viability assay (Annexin V): Cells were resuspended in Annexin V binding bufferer and stained with Alexa Fluor™ 350 Annexin V conjugate for 15min at RT in the dark before being analysed by FACS.

Instrument	FACS analysis: BD LSRFortessa™ X-20<br>FACS sorting: Aria 3 Sorter; BD
Software	BD FACSDiva™ used during FACS and FACS sorting<br>Additional analysis done using FLowJo
Cell population abundance	*Describe the abundance of the relevant cell populations within post-sort fractions, providing details on the purity of the samples and how it was determined.*
Gating strategy	For all samples, FSC/SSC gate was used for the starting cell population followed by doublets exclusion using FSC-W/FSC-A and/or SSC-W/SSC-A before gating for fluorescence markers.<br>NFKB2 knockdown samples: To sort for GFP positive cells, cells without GFP expression were used as a negative control to set a baseline. Cells detected to have a signal higher than the baseline were taken as GFP positive and collected.<br>Cell viability assay (Annexin V): unstained cells used as negative control to gate for Annexin V negative (live) cells.

☒ Tick this box to confirm that a figure exemplifying the gating strategy is provided in the Supplementary Information.

Magnetic resonance imaging

Experimental design

Design type	*Indicate task or resting state; event-related or block design.*
Design specifications	*Specify the number of blocks, trials or experimental units per session and/or subject, and specify the length of each trial or block (if trials are blocked) and interval between trials.*
Behavioral performance measures	*State number and/or type of variables recorded (e.g. correct button press, response time) and what statistics were used to establish that the subjects were performing the task as expected (e.g. mean, range, and/or standard deviation across subjects).*

Acquisition

Imaging type(s)	*Specify: functional, structural, diffusion, perfusion.*
Field strength	*Specify in Tesla*
Sequence & imaging parameters	*Specify the pulse sequence type (gradient echo, spin echo, etc.), imaging type (EPI, spiral, etc.), field of view, matrix size, slice thickness, orientation and TE/TR/flip angle.*
Area of acquisition	*State whether a whole brain scan was used OR define the area of acquisition, describing how the region was determined.*

Diffusion MRI ☐ Used ☐ Not used

Preprocessing

Preprocessing software	*Provide detail on software version and revision number and on specific parameters (model/functions, brain extraction, segmentation, smoothing kernel size, etc.).*
Normalization	*If data were normalized/standardized, describe the approach(es): specify linear or non-linear and define image types used for transformation OR indicate that data were not normalized and explain rationale for lack of normalization.*
Normalization template	*Describe the template used for normalization/transformation, specifying subject space or group standardized space (e.g. original Talairach, MNI305, ICBM152) OR indicate that the data were not normalized.*
Noise and artifact removal	*Describe your procedure(s) for artifact and structured noise removal, specifying motion parameters, tissue signals and physiological signals (heart rate, respiration).*
Volume censoring	*Define your software and/or method and criteria for volume censoring, and state the extent of such censoring.*

Statistical modeling & inference

Model type and settings	*Specify type (mass univariate, multivariate, RSA, predictive, etc.) and describe essential details of the model at the first and second levels (e.g. fixed, random or mixed effects; drift or auto-correlation).*
Effect(s) tested	*Define precise effect in terms of the task or stimulus conditions instead of psychological concepts and indicate whether ANOVA or factorial designs were used.*

Specify type of analysis: ☐ Whole brain ☐ ROI-based ☐ Both

Statistic type for inference

(See Eklund et al. 2016)

Specify voxel-wise or cluster-wise and report all relevant parameters for cluster-wise methods.

Correction

Describe the type of correction and how it is obtained for multiple comparisons (e.g. FWE, FDR, permutation or Monte Carlo).

Models & analysis

n/a	Involved in the study
☐	☐ Functional and/or effective connectivity
☐	☐ Graph analysis
☐	☐ Multivariate modeling or predictive analysis

Functional and/or effective connectivity

Report the measures of dependence used and the model details (e.g. Pearson correlation, partial correlation, mutual information).

Graph analysis

Report the dependent variable and connectivity measure, specifying weighted graph or binarized graph, subject- or group-level, and the global and/or node summaries used (e.g. clustering coefficient, efficiency, etc.).

Multivariate modeling and predictive analysis

Specify independent variables, features extraction and dimension reduction, model, training and evaluation metrics.
